# Supplementary material for: Biomimetic Grooved Ribbon Aerogel Inspired by the Structure of Pinus sylvestris var. mongolica Needles for Efficient Air Purification
Source: Polymers (Basel). 2025 Apr 30;17(9):1234. doi: 10.3390/polym17091234 (PMC12073558; doi:10.3390/polym17091234)
Supplement: Supplementary file 1 [file polymers-17-01234-s001.zip › polymers-3596964-supplementary.pdf]

Supporting Information

Supplementary Information contains:

Equation S1

Supplementary Figures S1−S4

Supplementary Table S1−S2

Equation S1

Doping content of UiO-66-NH_2 in UPG-x could calculate by Eq. (1) and Eq. (2) [36].

$$m_1 + m_2 = m \quad (1)$$

$$43.9\% \times m_1 + 21.4\% \times m_2 = y\% \times m \quad (2)$$

$$D = \frac{m_1}{m} \times 100\% \quad (3)$$

Where m_1, m_2 (mg) represent the weight of UiO-66-NH_2 and PG in the aerogels when the initial weight of UPG-x is m (mg), $y\%$ represent the residual weight percentage of UPG-5, UPG-10, UPG-15, and UPG-20 (22.7%, 23.6%, 24.5%, 26.3%) when 800 ◦C was selected as terminus, D represent the actual doping content. In addition, the residual weight percentage of UiO-66-NH_2 and PG was 43.9% and 21.4% when 800 ◦C was selected as terminus.

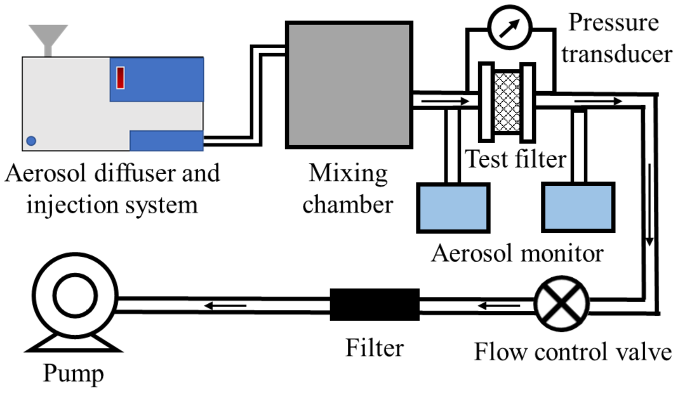

Figure S1. Test platform for assessing filtration performance.

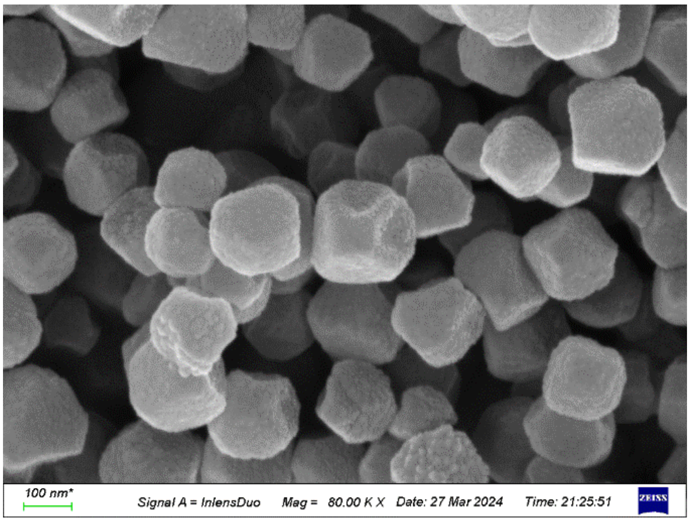

Figure S2. SEM of UiO-66-NH_2 nanoparticles.

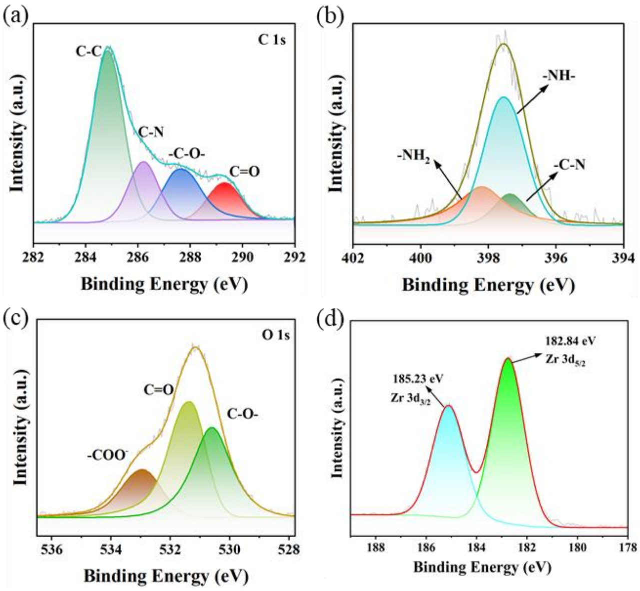

Figure S3. XPS spectra for the C, O, N and Zr region of UPG-10 aerogel.

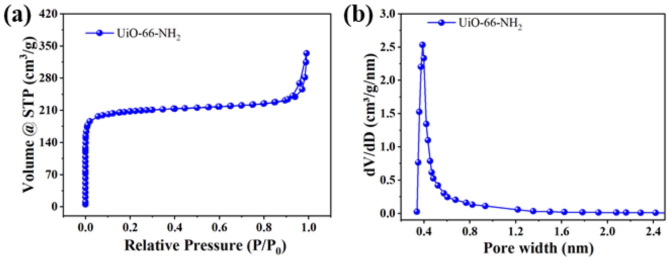

Figure S4. Nitrogen adsorption-desorption isotherms (a) and pore size distribution (b) of UiO-66-NH_2.

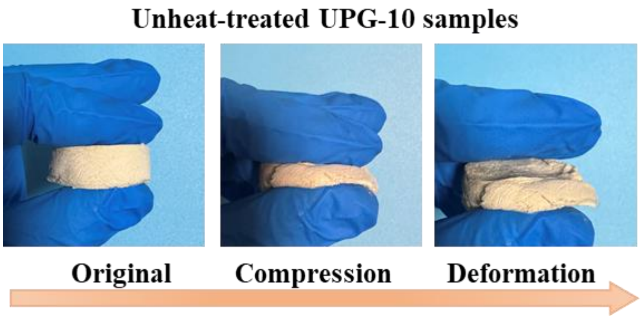

Figure S5. Non-heat-treated UPG aerogel demonstrated by (a) pressing test.

Table S1. The elemental composition of the aerogels.

Sample	Atomic (%)				
	C	O	N	Zr	Total (%)
UiO-66-NH_2	84.01	12.06	2.33	1.6	100
PG	66.47	25.09	8.44	0	100
UPG-5	62.57	29.13	7.86	0.44	100
UPG-10	63.49	28.4	7.37	0.74	100
UPG-15	64.2	27.62	7.13	1.05	100
UPG-20	66.48	25.42	6.94	1.16	100

Table S2. Composition of UPG-x.

Sample	UiO-66-NH_2 weight (mg)	PG weight (mg)	UiO-66-NH_2 ratio in suspension (wt.%)	The actual doping content (wt.%)
PG	0	50	0	0
UPG-5	2.5	50	4.8	5.8
UPG-10	5	50	9.1	9.8
UPG-15	7.5	50	13.0	13.8
UPG-20	10	50	16.7	21.8
